# Efficacy and safety of tocilizumab in patients with refractory generalized myasthenia gravis

**DOI:** 10.1111/cns.14793

**Published:** 2024-06-18

**Authors:** Zhe Ruan, Yonglan Tang, Ting Gao, Chunhong Li, Rongjing Guo, Chao Sun, Xiaoxi Huang, Zhuyi Li, Ting Chang

**Affiliations:** ^1^ Department of Neurology, Tangdu Hospital The Fourth Military Medical University Xi'an China

**Keywords:** conventional immunotherapy, generalized estimating equation, generalized myasthenia gravis, interleukin 6 receptor, tocilizumab

## Abstract

**Background:**

We aimed to compare the efficacy of tocilizumab with conventional immunotherapy in refractory patients with acetylcholine receptor antibody‐positive (AChR‐Ab+) generalized myasthenia gravis (gMG).

**Methods:**

This single‐center prospective cohort study was based on patients from an MG registry study in China and conducted from February 10, 2021 to March 31, 2022. Adult refractory patients with AChR‐Ab+ gMG were assigned to tocilizumab or conventional immunotherapy groups. The primary efficacy outcome was the mean difference of MG activities of daily living (MG‐ADL) change at weeks 4, 8, 12, 16, 20, 24 corresponding to that at the baseline between the two groups. A generalized estimating equation model was used for the primary outcome analysis. Safety was assessed based on adverse events.

**Results:**

Of 34 eligible patients, 20 (mean [standard deviation] age, 53.8 [21.9] years; 12 [60.0%] female) received tocilizumab and 14 received conventional immunotherapy (45.8 [18.0] years; 8 [57.1%] female). The tocilizumab group had greater reduction in MG‐ADL score at week 4 (adjusted mean difference, −3.4; 95% CI, −4.7 to −2.0; *p* < 0.001) than the conventional immunotherapy group, with significant differences sustained through week 24 (adjusted mean difference, −4.5; 95% CI, −6.4 to −2.6; *p* < 0.001). At week 24, the proportion of patients achieving higher levels of MG‐ADL (up to 7‐point reduction) and QMG (up to 11‐point reduction) scores improvement was significantly greater with tocilizumab. Tocilizumab had acceptable safety profiles without severe or unexpected safety issues.

**Conclusion:**

Tocilizumab is safe and effective in improving the MG‐ADL score and reducing prednisone dose in refractory AChR‐Ab+ gMG, suggesting tocilizumab has the potential to be a valuable therapeutic option for such patients.

## Graphical abstract

This study encompassed a cohort of 34 patients diagnosed with myasthenia gravis (MG), who were administered either tocilizumab or conventional immunotherapy. The findings of this research indicate that tocilizumab exhibits prompt amelioration of symptoms associated with generalized myasthenia gravis (gMG), suggesting its potential as a viable therapeutic approach for managing this condition.

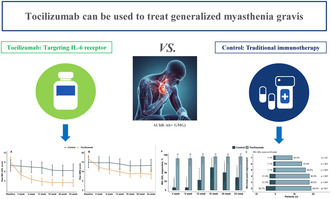

## 1 INTRODUCTION

Myasthenia gravis (MG) is an acquired autoimmune disease caused by acetylcholine receptor (AChR) antibodies in approximately 80% of cases.^1^ Most patients can be successfully managed with nonspecific immunotherapies, such as corticosteroid and non‐steroidal immunosuppressive treatments (NSIST). However, the adverse events (AEs) of long‐term nonspecific immunotherapies remain a hurdle for MG treatment. A case–control study reported that around 48.2% of patients with MG expressed concerns regarding short‐ and long‐term treatment AEs and that 55.8% discontinued treatment due to AEs associated with current therapies.^2^ Despite these therapies, MG may not be well controlled or refractory due to an incomplete response or poor tolerance.

Recently, there has been a surge in the development of new therapies targeting B and T cells, component 5 (C5), neonatal Fc receptor (FcRn), and cytokines. These promising biological drugs improved MG symptoms and were well tolerated in clinical trials, leading to their approval or extension to phase II and III trials.^3^, ^4^, ^5^, ^6^, ^7^, ^8^

Interleukin 6 (IL‐6), a keystone cytokine, is involved in the survival, expansion, and maturation of B cells and plasmablasts, as well as the production of autoantibodies, making it a potential candidate for MG treatment. Studies on experimental autoimmune MG (EAMG) have shown that clinical symptoms can be reversed using anti‐IL‐6 antibodies. Suppression of EAMG by anti‐IL‐6 antibodies was accompanied by a decrease in the overall rat anti‐AChR antibody titer and a reduced number of B cells compared with the control treatment.^9^ Additionally, the disruption of IL‐6 signaling may switch immune responses in vivo from the induction of regulatory T cells to pathogenic IL‐17‐producing T helper cells.^10^ Currently, a study evaluating the efficacy and safety of satralizumab (an IL‐6 receptor blocking agent) as an add‐on therapy in patients with gMG is ongoing. Nevertheless, the efficacy of targeted IL‐6 treatment for MG remains unsupported by empirical evidence.

Tocilizumab, the first humanized anti‐IL‐6 receptor monoclonal antibody (mAb), has been approved for the treatment of rheumatoid arthritis (RA) and juvenile idiopathic arthritis and is now being investigated in neuromyelitis optica spectrum disorder (NMOSD), with promising results in preventing disease relapse.^11^ Data from long‐term cumulative safety analysis demonstrated that tocilizumab had a safety profile in the treatment of patients with RA.^12^ Although no studies have been performed yet, case reports of refractory MG responding favorably to tocilizumab suggest its possible efficacy in MG.^13^

Thus, this study aimed to compare the efficacy and safety of tocilizumab with those of conventional immunotherapy in refractory patients with AChR antibody‐positive generalized MG (AChR‐Ab+ gMG).

## 2 METHODS

### 2.1 Study design

This single‐center prospective cohort study, which was based on patients from an MG registry study (https://neuroreg.se/) in China that covered more than 2000 patients with MG, was approved by the Ethics Committee of Tangdu Hospital, Fourth Military Medical University (202102‐06). All the patients provided written informed consent for registration in the Tangdu MG registry. Patients receiving tocilizumab provided independent written informed consent before treatment and were not incentivized to participate in this study. This study followed the Strengthening the Reporting of Observational Studies in Epidemiology reporting guidelines for cohort studies and was registered with the Chinese Clinical Trial Registry (ChiCTR2100043273).

### 2.2 Participants

We identified patients in the MG registry study who received tocilizumab or conventional immunotherapy (i.e., prednisone, azathioprine, tacrolimus, cyclosporin A, and mycophenolate mofetil) between February 10, 2021 and September 30, 2021. Adult refractory patients with AChR‐Ab+ gMG were eligible for inclusion. Patients with refractory MG were defined as the patients had an inadequate response (MG Post‐Intervention Status is unchanged or worse) to ≥1 immunosuppressants used in adequate doses for an adequate duration (including daily prednisone at doses ≥30 mg over a minimum of 3 months and 3 or more months treatment with azathioprine ≥150 mg/day, tacrolimus ≥3 mg/day, cyclosporin A ≥ 100 mg/day, and mycophenolate mofetil ≥1500 mg/day since disease onset), with persistent symptoms or side effects (including low blood cells, severe osteoporosis, femoral head necrosis, alopecia, obesity, gastrointestinal ulcers, tremor, psychiatric symptoms, etc.) that limit functioning defined by the patient and physician.^14^ Eligible patients should be categorized to have Myasthenia Gravis Foundation of America (MGFA) class II to IVa; and had a Quantitative Myasthenia Gravis (QMG) score of ≥11 or a Myasthenia Gravis Activities of Daily Living (MG‐ADL) score of ≥5 (with <50% of the score due to ocular symptoms).

The exclusion criteria were as follows: active hepatitis B, seropositivity for hepatitis C, seropositivity for human immunodeficiency virus with a low CD4 count, intravenous immunoglobulin (IVIg) or plasma exchange within 1 month of screening, receiving biologics such as rituximab and eculizumab within 6 months of screening, undergoing thymectomy within 6 months or planning to undergo thymectomy in the next 3 months of screening, and concurrent invasive thymoma.

### 2.3 Data collection

Demographic and clinical data were collected from the Tangdu MG registry database and validated against medical records. Anti‐AChR antibody was tested in all patients using a cell‐based assay (CBA) method, which was reported have a higher sensitivity and specificity than ELISA and radioimmunoassay methods.^15^ The following baseline data were collected: sex; age; age at onset; disease duration; body mass index; comorbid autoimmune diseases (e.g., the presence of systemic lupus erythematosus, multiple sclerosis, RA, Hashimoto's thyroiditis, and NMOSD); seropositive for anti‐titin antibody; and thymic status classified as normal, hyperplasia, thymoma, or other types based on radiology and pathological evaluations (patients who underwent thymectomy were evaluated based on pathology findings and those who did not undergo surgery were evaluated based on radiology findings); previous thymectomy; time from disease onset to surgery; MG crisis history; QMG, MG‐ADL, and Myasthenia Gravis Composite (MGC) scores; dose and duration of prednisone and NSIST previously and at enrollment; and previous treatment with biologics such as rituximab and eculizumab.

### 2.4 Interventions

Investigators grouped patients into the tocilizumab group or the control group based on whether they received tocilizumab treatment. The treatment schedule was determined by the patient and physician together. Investigators were responsible to follow up prospectively after achieving informed consent forms. Patients in the tocilizumab group received intravenous tocilizumab (8 mg/kg every 4 weeks) for 24 weeks and were allowed to keep their original treatment regimen including prednisone and NSIST. The dose and administration frequency of tocilizumab was based on the recommended treatment regimen for patients with RA. Patients in the control group received prednisone and/or NSIST. During the study period, the dose of corticosteroids or immunosuppressants was allowed to be adjusted based on the symptoms change and AEs at each visit. If the symptoms worsened, as defined by an MG‐ADL score of ≥2, the prednisone dose increased to 0.5 mg/kg/day until minimal manifestation status (MMS) was attained, and the maximum dose did not exceed 0.75–1 mg/kg/day. If the symptoms improved, the initial dose was maintained for 4–6 weeks; subsequently, the prednisone dose was reduced by 10 mg every 2 weeks until a dose of 20 mg was reached, with subsequent slowing of the tapering to 5 mg monthly. The prednisone dose was reduced in the same manner in cases of major steroid‐related AEs. Treatment information and drug‐related AEs were recorded at each visit.

### 2.5 Outcomes

The primary efficacy outcome was the mean difference in the MG‐ADL score change at each study visit (weeks 4, 8, 12, 16, 20, and 24), corresponding to that at the baseline between the two groups. Secondary outcomes included the following: (1) the mean difference in QMG and MGC score changes compared with that at the baseline; (2) the mean difference of daily prednisone dose compared with that at baseline; and (3) the proportion of MG‐ADL (defined as a ≥2‐point reduction in the total MG‐ADL score) and QMG responders (defined as a ≥3‐point reduction in the total QMG score).^16^, ^17^, ^18^ To minimize subjective differences among assessors, all patients' assessment was conducted by an independent assessor who was not involved in the patients' treatment. Additionally, we also established unified inquiry standards for each item of QMG to reduce the bias.

Safety was assessed based on AEs reported by investigators or participants. All AEs were recorded according to Common Terminology Criteria for Adverse Events (version 5.0).

### 2.6 Follow‐up

Assessments were performed every 4 weeks for up to 24 weeks. All participants in the tocilizumab group underwent face‐to‐face visits for 24 weeks. Six participants in the control group failed to perform face‐to‐face visits at 4 or 8 weeks owing to the impact of COVID‐19 pandemic. We performed visits by video using a uniform case report form and collected MG‐ADL scores and treatment feedback. To avoid the effect of video follow‐up on the collected data, the same neurologist performed the assessment with a unified questionnaire that was used as face‐to‐face visit.

### 2.7 Statistical analyses

We calculated the sample size using a repeated measures design by comparing the means of the two groups.^19^, ^20^ As a preliminary study, there were no relevant data available for reference, so we calculated the sample size according to the results of other clinical trials in MG as well as conservative intraclass correlations. The planned sample size of 20 participants can provide 84% power to detect a difference between the two groups on the primary efficacy outcome (mean change in MG‐ADL score from the baseline to week 24). A design with 7‐times repeated measures and a compound symmetry covariance structure were employed. Assuming a 4 mean difference of MG‐ADL and a standard deviation of 4 between the two groups, and the intraclass correlation coefficient was 0.5 and an alpha level was 0.05.

We conducted normality tests on all continuous variables using the Shapiro–Wilk test. Continuous variables were presented as the mean (standard deviation) for normally distributed data and median (interquartile range) for non‐normally distributed variables. Quantitative data are presented as numbers (percentages). Baseline characteristics between the two groups were compared using the Student's *t*‐test or Mann–Whitney *U*‐test for continuous variables and Fisher's exact test for categorical variables. A generalized estimating equation (GEE) model was used for the primary outcome analysis. Baseline MG‐ADL, QMG, and MGC scores and daily prednisone dose were separately included in the GEE model as covariates. A pairwise comparison was performed to compare the mean differences in the MG‐ADL, QMG, and MGC scores and daily prednisone dose at each study visit between the two groups. The proportions of MG‐ADL and QMG responders were analyzed using Fisher's exact test. In addition, MG‐ADL and QMG responders were redefined based on different improvement threshold points (a ≥3‐, 4‐, 5‐, 6‐, or 7‐point reduction in the MG‐ADL score and ≥6‐, 7‐, 8‐, 9‐, 10‐, or 11‐point reduction in the QMG score) and compared between the two groups at week 24. There were no missing data for the MG‐ADL. The missing data mainly associated with QMG and MGC, and a recorded QMG and MGC was available for 200 of 206 total visits (97.1%). The missing data, accounting for 2.9%, was not imputed by multiple imputation approach, because GEE model can address the issue of randomly missing data.^21^

Sensitivity analyses for the primary and secondary outcomes were performed by introducing different covariates and factors into the GEE model. Three models were constructed. The full model included group‐time‐interaction effects, baseline measures of outcomes, and other baseline variables; the simplified model included group‐time‐interaction effects, baseline measures of outcomes, and variables with significant fixed effects of the full model; and the final model included group‐time‐interaction effects and baseline measures of outcomes.

*p* Values of <0.05 were considered statistically significant. All analyses were performed using *R* 4.13 (R Foundation for Statistical Computing), and the packages used in the analyses are shown in Table S1.

### 2.8 Role of the funding source

The funders of the study had no role in study design, data collection, data analysis, data interpretation, or writing of the report.

## 3 RESULTS

### 3.1 Demographic and clinical characteristics

**FIGURE 1:**
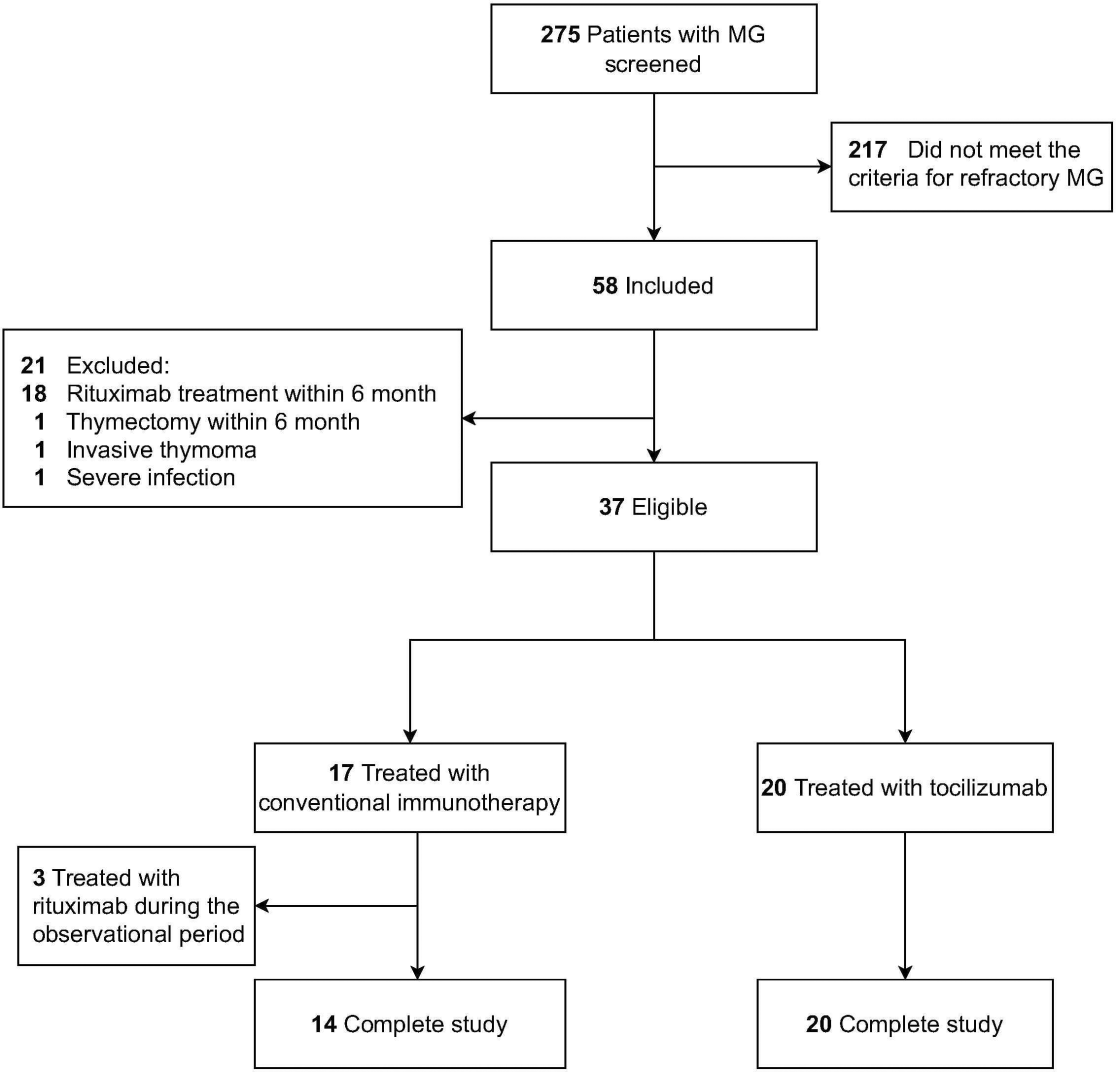
Study flowchart showing the selection process for patients with MG. Three patients whose treatment was switched to rituximab after conventional immunotherapy failed withdrew from the study in the control group. MG, myasthenia gravis.

Overall, 275 patients with MG were registered during the study period, among whom 37 fulfilled the inclusion and exclusion criteria. Three patients in the control group withdrew from the study due to receiving rituximab; thus, 34 patients were included in the final analysis (Figure 1). The individual immunotherapy courses of the 34 patients are shown in Table S2.

Of the 34 patients, 20 (58.8%) were female individuals. The age was 50.5 (20.5) years, while the age at onset was 46.7 (20.8) years. The disease duration was 36.5 (18.3, 61.8) months. Twenty (including 12 female) patients received tocilizumab, while 14 (including 8 female) patients received conventional immunotherapy. Patients in the tocilizumab group had slightly worse symptoms than those in the control group, which was reflected by the MG‐ADL score (mean 9.0 vs. 7.9), QMG score (mean 16.7 vs. 15.0) and MGC score (mean 16.8 vs. 15.6) but did not reach statistical significance. No significant differences were found in daily prednisone dose between tocilizumab and control group (mean 25.0 ± 14.1 vs. 26.4 ± 12.6 mg, *p* = 0.76). In each of the two groups, two patients had previously undergone rituximab administration at a dosage of 500 mg, with a frequency of once every 6–8 months, and both individuals received more than two treatment cycles. Despite these interventions, the desired treatment outcome was not achieved (Table S2). There were no significant differences observed in any other baseline characteristics between the two groups (Table 1).

**TABLE 1:** Baseline demographic and clinical characteristics of the patients.

Characteristics	Overall (*n* = 34)	Control (*n* = 14)	Tocilizumab (*n* = 20)	*p‐*Value
Sex, no. (%)				
Female	20 (58.8)	8 (57.1)	12 (60.0)	0.99
Male	14 (41.2)	6 (42.9)	8 (40.0)	
Age, mean (SD), years	50.5 (20.5)	45.8 (18.0)	53.8 (21.9)	0.27
Age of onset, years, no. (%)				
Early‐onset MG <50	13 (38.2)	7 (50.0)	6 (30.0)	0.30
Late‐onset MG ≥50	21 (61.8)	7 (50.0)	14 (70.0)	
Age of onset, years, mean (SD)	46.7 (20.8)	42.5 (18.0)	49.6 (22.5)	0.34
Disease duration, months, median (IQR)	36.5 [18.3, 61.8]	34.5 [22.8, 69.8]	37.5 [14.8, 61.3]	0.93
BMI, mean (SD)	24.2 (3.5)	24.9 (4.3)	23.8 (2.9)	0.35
MGFA class, no. (%)				
II	13 (38.2)	8 (57.1)	5 (25.0)	0.10
III	14 (44.1)	3 (21.4)	11 (55.0)	
IV	7 (17.6)	3 (21.4)	4 (20.0)	
Comorbid AD^a^, no. (%)				
No	25 (82.4)	11 (78.6)	14 (70.0)	0.70
Yes	9 (17.6)	3 (21.4)	6 (30.0)	
Anti‐titin antibody, no. (%)				
Negative	12 (44.4)	5 (45.5)	7 (43.8)	0.99
Positive	15 (55.6)	6 (54.5)	9 (56.2)	
Thymic status, no. (%)				
Normal	20 (58.8)	7 (50.0)	13 (65.0)	0.53
Hyperplasia	7 (20.6)	3 (21.4)	4 (20.0)	
Thymic Cyst	1 (2.9)	0 (0.0)	1 (5.0)	
Thymoma	6 (17.6)	4 (28.6)	2 (10.0)	
Previous thymectomy, no. (%)				
No	25 (73.5)	9 (64.3)	16 (80.0)	0.44
Yes	9 (26.5)	5 (35.7)	4 (20.0)	
Time from disease onset to surgery, months, median (IQR)	11.0 [3.0, 22.0]	11.0 [6.0, 22.0]	9.5 [2.5, 24.0]	0.81
MG crisis history, no. (%)				
No	22 (64.7)	8 (57.1)	14 (70.0)	0.49
Yes	12 (35.3)	6 (42.9)	6 (30.0)	
MG‐ADL score, mean (SD)	8.5 (3.4)	7.9 (3.1)	9.0 (3.6)	0.39
QMG score, mean (SD)	16.0 (4.7)	15.0 (5.2)	16.7 (4.2)	0.30
MGC score, mean (SD)	16.3 (7.9)	15.6 (8.4)	16.8 (7.8)	0.70
Daily prednisone dose, mg, mean (SD)	25.6 (13.3)	26.4 (12.6)	25.0 (14.1)	0.76
Previous NSIST^b^				
AZA	12	7	5	–
MMF	7	2	5	
TAC	20	7	13	
CA	1	1	0	
Previous biologic agents				
Rituximab	4	2	2	
NSIST at enrolment, no. (%)				
AZA	5 (14.7)	2 (14.3)	3 (15.0)	0.40
MMF	3 (8.8)	2 (14.3)	1 (5.0)	
TAC	15 (44.1)	7 (50.0)	8 (40.0)	
CA	1 (2.9)	1 (7.1)	0 (0.0)	
None	10 (29.4)	2 (14.3)	8 (40.0)	

Abbreviations: AD, autoimmune disease; AZA, Azathioprine; CA, Cyclosporine A; CI, confidence interval; IQR, interquartile range; MG, myasthenia gravis; MG‐ADL, Myasthenia Gravis Activities of Daily Living; MGC, Myasthenia Gravis Composite; MGFA, Myasthenia Gravis Foundation of America; MMF, Mycophenolate mofetil; NSIST, Non‐steroidal immunosuppressive treatments; QMG, Quantitative Myasthenia Gravis; TAC, Tacrolimus.

^a^ Included Hashimoto thyroiditis (7 patients), RA (1 patient) and undifferentiated connective tissue disease (1 patient).

^b^ Eight patients previously used two NSIST and one patient used three NSIST.

### 3.2 Efficacy

**FIGURE 2:**
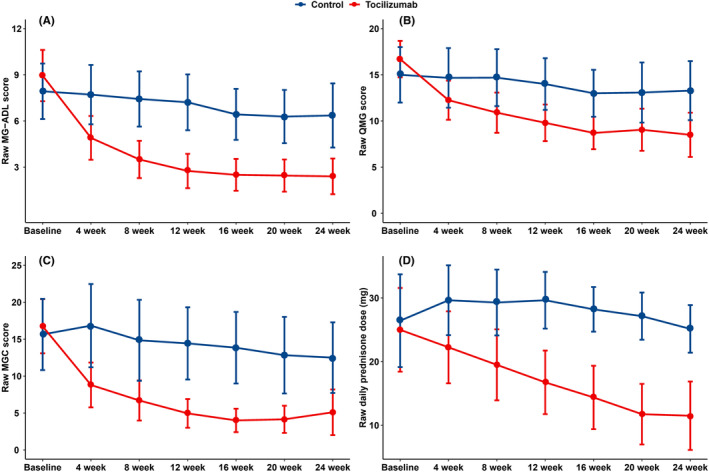
Mean changes in MG‐ADL (A), QMG (B), MGC (C) scores, and the daily prednisone dose (D) from the baseline to 24 weeks in the tocilizumab and control groups. The line charts show mean change in all scores measure and the daily prednisolone dose from the baseline to 24 weeks in the tocilizumab and control groups with data points showing mean scores and error bars showing 95% confidence interval of mean scores. MG‐ADL, myasthenia gravis activities of daily living; MGC, myasthenia gravis composite; QMG, quantitative myasthenia gravis.

Interactions were observed for the MG‐ADL scores (*p* = 0.01) in the group‐by‐time interaction analysis. Compared with patients in the control group, those in the tocilizumab group had a greater reduction in the MG‐ADL score at week 4 (adjusted mean difference, −3.4; 95% confidence interval [CI], −4.7 to −2.0; *p* < 0.001); this significant difference sustained until week 24 (adjusted mean difference, −4.5; 95% CI, −6.4 to −2.6; *p* < 0.001). A significant reduction in the QMG score in the tocilizumab group was observed at week 8 (adjusted mean difference, −4.6; 95% CI, −6.8 to −2.4; *p* < 0.001) and sustained until week 24 (adjusted mean difference, −5.8; 95% CI, −9.2 to −2.4; *p* = 0.004). Patients in the tocilizumab group had a greater reduction in the MGC score at week 4 (adjusted mean difference, −7.9; 95% CI, −11.2 to −4.6; *p* < 0.001), which was sustained until week 24 (adjusted mean difference, −8.0; 95% CI, −12.5 to −3.5; *p* = 0.002; Table 2; Figure 2). The individual change in the MG‐ADL, QMG, and MGC scores as well as daily prednisone dose from the baseline to 24 weeks are shown in Figure S1. It was noted that individual scores were significantly higher at two follow‐ups, especially at week 24. The reason for this phenomenon was two young women in the tocilizumab group had transient worsening of symptoms during menstruation. At week 24, these two patients had menstruating, resulting in higher QMG and MGC scores.

**TABLE 2:** Efficacy for the primary and secondary outcomes by generalized estimated equation analysis.

Outcome	Control (*n* = 14)	Tocilizumab (*n* = 20)	Group‐by‐time interaction effect	Group effect	Time effect	Tocilizumab vs. control	
						Adjusted mean difference (95% CI)^a^	*p*‐Value
MG‐ADL score, mean (95 CI%)							
Baseline	7.9 (6.1 to 9.7)	9.0 (7.3 to 10.6)	0.01	<0.001	<0.001	–	–
Week 4	7.7 (5.8 to 9.6)	4.9 (3.5 to 6.3)				−3.4 (−4.7 to −2.0)	<0.001
Week 8	7.4 (5.6 to 9.2)	3.5 (2.3 to 4.7)				−4.5 (−5.7 to −3.2)	<0.001
Week 12	7.2 (5.4 to 9.0)	2.8 (1.6 to 3.9)				−5.0 (−6.4 to −3.6)	<0.001
Week 16	6.4 (4.8 to 8.1)	2.5 (1.5 to 3.5)				−4.5 (−5.7 to −3.2)	<0.001
Week 20	6.3 (4.6 to 8.0)	2.5 (1.4 to 3.5)				−4.4 (−5.9 to −2.9)	<0.001
Week 24	6.4 (4.3 to 8.4)	2.4 (1.2 to 3.6)				−4.5 (−6.4 to −2.6)	<0.001
QMG score, mean (95 CI%)							
Baseline	15.0 (12.0 to 18.0)	16.7 (14.7 to 18.7)	0.05	0.008	<0.001	–	–
Week 4	14.7 (11.4 to 17.9)	12.2 (10.1 to 14.4)				−2.8 (−5.1 to −0.6)	0.09
Week 8	14.7 (11.6 to 17.8)	10.9 (8.7 to 13.1)				−4.6 (−6.8 to −2.4)	<0.001
Week 12	14.0 (11.2 to 16.8)	9.8 (7.8 to 11.8)				−5.2 (−7.4 to −3.0)	<0.001
Week 16	13.0 (10.5 to 15.5)	8.7 (6.9 to 10.5)				−5.3 (−7.1 to −3.4)	<0.001
Week 20	13.1 (9.8 to 16.3)	9.1 (6.8 to 11.3)				−5.4 (−8.5 to −2.4)	0.004
Week 24	13.3 (10.1 to 16.5)	8.5 (6.1 to 10.9)				−5.8 (−9.2 to −2.4)	0.004
MGC score, mean (95 CI%)							
Baseline	15.6 (10.8 to 20.5)	16.8 (13.1 to 20.4)	0.26	<0.001	<0.001	–	–
Week 4	16.8 (11.2 to 22.5)	8.8 (5.8 to 11.8)				−7.9 (−11.2 to −4.6)	<0.001
Week 8	14.8 (9.4 to 20.3)	6.7 (4.0 to 9.4)				−8.5 (−11.7 to −5.2)	<0.001
Week 12	14.4 (9.5 to 19.3)	5.0 (3.0 to 6.9)				−10.1 (−13.1 to −7.0)	<0.001
Week 16	13.8 (9.0 to 18.7)	4.0 (2.4 to 5.6)				−10.2 (−13.0 to −7.4)	<0.001
Week 20	12.8 (7.6 to 18.0)	4.2 (2.3 to 6.0)				−9.2 (−12.5 to −5.8)	<0.001
Week 24	12.5 (7.7 to 17.3)	5.1 (2.0 to 8.2)				−8.0 (−12.5 to −3.5)	0.002
Daily prednisone dose, mg, mean (95 CI%)							
Baseline	26.4 (19.1 to 33.7)	25.0 (18.4 to 31.6)	0.27	<0.001	0.002	–	–
Week 4	29.6 (24.2 to 35.1)	22.2 (16.6 to 27.9)				−6.8 (−11.0 to −2.7)	0.007
Week 8	29.3 (24.1 to 34.5)	19.5 (13.9 to 25.1)				−9.2 (−14.3 to −4.2)	0.002
Week 12	29.6 (25.2 to 34.1)	16.8 (11.8 to 21.7)				−12.3 (−17.7 to −6.9)	<0.001
Week 16	28.2 (24.7 to 31.7)	14.4 (9.4 to 19.4)				−13.3 (−18.7 to −7.9)	<0.001
Week 20	27.1 (23.4 to 30.8)	11.8 (7.0 to 16.5)				−14.8 (−20.7 to −9.0)	<0.001
Week 24	25.1 (21.4 to 28.9)	11.5 (6.1 to 16.9)				−13.1 (−19.4 to −6.7)	<0.001

Abbreviations: CI, confidence interval; MG‐ADL, Myasthenia Gravis Activities of Daily Living; MGC, Myasthenia Gravis Composite; QMG, Quantitative Myasthenia Gravis.

^a^ GEE model separately included the baseline MG‐ADL, QMG, MGC scores and daily prednisone dose as covariates.

The daily prednisone dose was reduced more significantly in the tocilizumab group (adjusted mean difference, −6.8 mg; 95% CI, −11.0 to −2.7; *p* = 0.007) at 4 week and sustained until 24 weeks (adjusted mean difference, −13.1 mg; 95% CI, −19.4 to −6.7; *p* < 0.001; Table 2; Figure 2). The adjusted means of all score measures and daily prednisone dose calculated using the GEE are shown in Table S3 and Figure S2.

### 3.3 Sensitivity analyses

**FIGURE 3:**
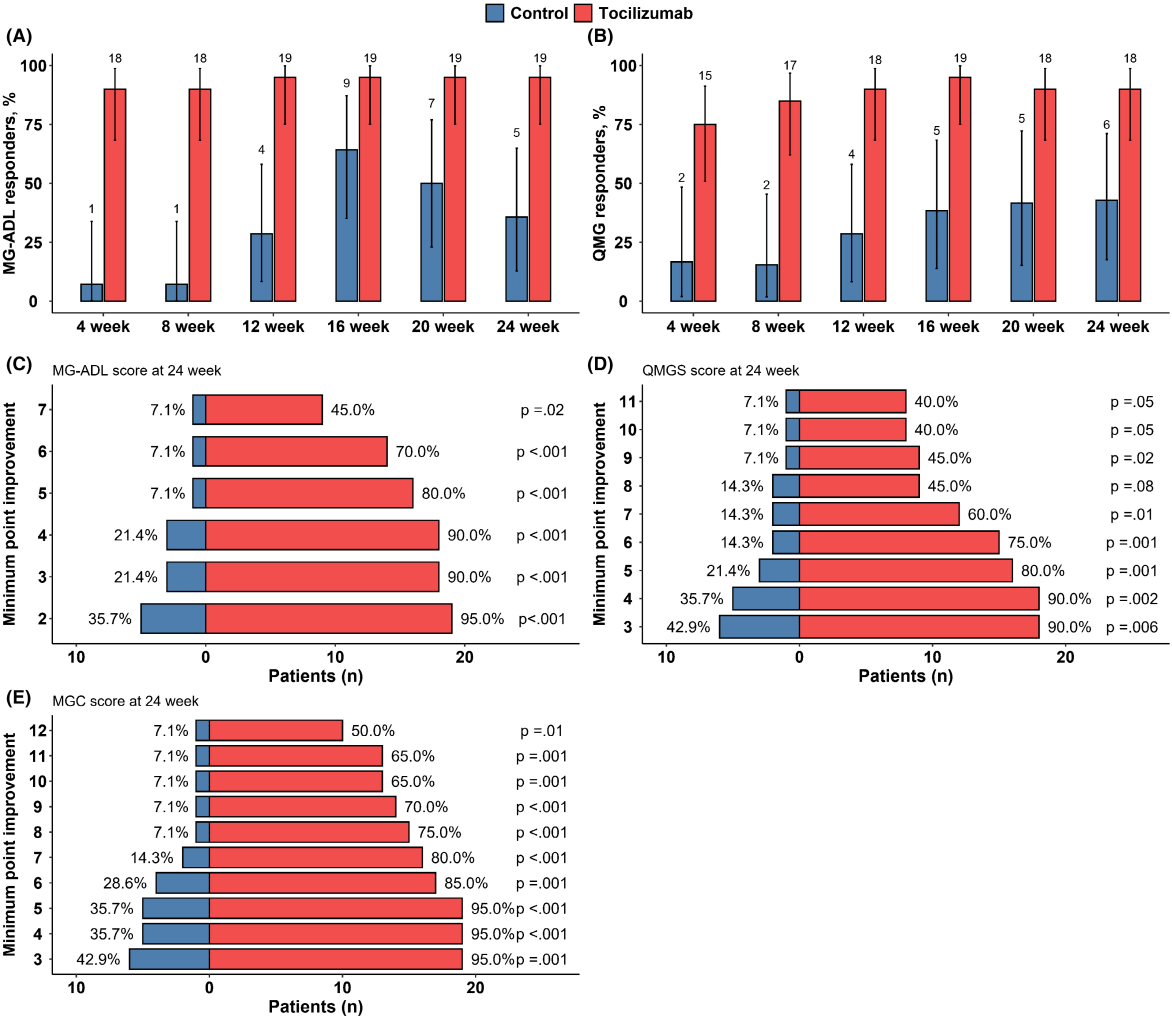
Sensitivity analyses. Proportion of the MG‐ADL responders (A) and the QMG responders (B) form week 4 at week 24 in the tocilizumab and control groups. Minimum point improvement in MG‐ADL (C), QMG (D), and MGC (E) scores at 24 weeks from the start of therapy. MG‐ADL, myasthenia gravis activities of daily living; MGC, myasthenia gravis composite; QMG, quantitative myasthenia gravis.

In the sensitivity analyses, the proportions of MG‐ADL and QMG responders from week 4 to 24 were larger in the tocilizumab group than in the control group (95.0% vs. 35.7% in the MG‐ADL score, 90.0% vs. 42.9% in the QMG score at week 24, *p* < 0.001; Table S4; Figure 3A,B). Additionally, the proportion of patients with higher levels of improvement in the MGADL, QMG, and MGC scores was significantly greater in the tocilizumab group at week 24 (up to a 7‐point reduction in the MG‐ADL, 45% vs. 7.1%, *p* = 0.02; up to an 11‐point reduction in the QMG, 40% vs. 7.1%, *p* = 0.05; up to a 12‐point reduction in the MGC, 50% vs. 7.1%, *p* = 0.01; Figure 3C–E).

The results of GEE analyses showed that group, time, and group‐by‐time interaction effects were observed in the full, simplified, and final models, and the adjusted mean differences of the MG‐ADL, QMG, and MGC scores and daily prednisone dose changes were consistent with the main outcomes (Tables S5–S8).

### 3.4 Safety

Tocilizumab showed acceptable safety profiles, with a total of 10 tocilizumab‐related AEs. All were graded 1/2 and manageable. Details of the AEs are shown in Table S9.

## 4 DISCUSSION

Our findings showed that tocilizumab provided rapid and clinically and statistically meaningful symptom improvement and steroid dose reduction with a favorable safety profile. Symptom improvement with tocilizumab was observed from week 4 and sustained until week 24. At week 24, approximately 95% of the patients in the tocilizumab group achieved improved outcomes beyond the clinically meaningful threshold, showing a ≥2‐point reduction in the MG‐ADL score (vs. 35.7% with conventional immunotherapy). To the best of our knowledge, this is the first study to compare the efficacy and safety of tocilizumab with those of conventional immunotherapy for the treatment of refractory patients with AChR‐Ab+ gMG, providing new insights into the possibility of treating MG with IL‐6 receptor blockers.

Currently, advances in MG treatment have led to a marked reduction in disease‐associated mortality, however, an unmet need still exists for the management of the disease especially for the refractory cases.^22^ Recently, innovative drugs have emerged as important therapeutic tools that promise to change the treatment algorithm for MG. For instance, the benefit of treatment with rituximab, a B cell depletion therapy, has been established for the treatment of refractory AChR‐Ab+ gMG and muscle‐specific tyrosine kinase (MuSK)‐MG in several studies.^23^, ^24^, ^25^, ^26^, ^27^, ^28^ The RINOMAX trial found that a single low rituximab dose was clinically effective for newly diagnosed gMG.^29^ With two reports of new therapies for MG targeting FcRn and C5,^4^, ^5^ a new era for the treatment of this autoimmune disease is emerging.

Reportedly, IL‐6 drives the pathogenesis of MG, which is supported by the link between IL‐6 and B cells and follicular helper T (TFH) cells.^30^ Previous studies have identified IL‐6 as a promoter of the differentiation and proliferation of B cells and an inducer of the maturation of B cells into antibody‐producing plasma cells, as well as the generation of TFH cells. Theoretically, the blockage of IL‐6 is supposed to suppress various ongoing autoimmune diseases.^31^ Tocilizumab, a humanized IL6‐receptor mAb, can reduce the risk of NMOSD relapse as compared to azathioprine. In patients with RA when rituximab failed despite adequate B cell depletion, IL‐6‐directed therapy may be an effective treatment choice.^32^ Satralizumab, a pharmacologically optimized IL6‐receptor mAb, has been approved for the treatment of NMOSD.^33^ A phase III clinical trial aiming to evaluate the effectiveness and safety of satralizumab in MG is still ongoing (NCT04963270). NMOSD and MG are mediated by pathogenic autoantibodies of AQP4 and NMJ postsynaptic proteins (AChR, MuSK, and LRP4), respectively. These two diseases share some similar pathogenic mechanisms including the breakdown of tolerance, the collaboration of T cells and B cells, imbalances in T helper 1 (Th1)/Th2/Th17/regulatory T cells, and complement system activation.

A prospective, open‐label, single‐arm study has evaluated the efficacy and safety in patients with AChR+ gMG in a 48‐week follow‐up study.^34^ Tocilizumab was associated with a good clinical response and safety, as evidenced by significant improvements in QMG score (from 15.5 at the baseline to 4 at week 12) and MG‐ADL score (from 14.5 at the baseline to 4 at week 12), decreased AChR antibody titers (from 15 at the baseline to 6.8 at week 12), and discontinued prednisone without serious AEs. These findings suggest that IL‐6 receptor could be a therapeutic target for MG, although future studies involving larger sample size and a control group are still required.

Current rapid‐acting treatments in MG include IVIg administration, plasma exchange, and steroid use. Tocilizumab has a rapid onset of action, with efficacy observed as early as week 4 in our study, making it a potential first‐line treatment option that may be able to partially address the limitations of plasma supply and AEs because of steroid use. Additionally, tocilizumab led to a remarkable symptom improvement compared with conventional immunotherapy. Many patients had improvement beyond the clinically meaningful threshold, achieving up to 7‐point reductions in MGADL and 11‐point reductions in QMG. As an add‐on treatment to baseline immunosuppressants, tocilizumab was shown to make the process of prednisone reduction faster. It is noteworthy two patients in the cohort received tocilizumab monotherapy because of treatment‐resistance or intolerance to the side effects of conventional drugs. Their symptoms improved greatly with MG‐ADL reducing from 12 at the baseline to 3 at week 24 and from 13 at the baseline to 5 at week 24, respectively, suggesting the efficacy of tocilizumab monotherapy. In multiple sclerosis, an earlier start of effective treatment is associated with reduced damage to neuro‐axonal connections.^35^ In MG, low‐dose rituximab showed efficacy in some patients with generalized AChR‐gMG, especially if started early after diagnosis.^29^ It is warranted to further explore the efficacy of tocilizumab monotherapy and of initiation in early‐stage.

The reported AEs associated with tocilizumab in our study were consistent with its known safety profile in RA.^36^, ^37^, ^38^ The long‐term safety profile of tocilizumab (mean treatment duration, 2.4 years) from several phase III trials and open‐label extensions did not demonstrate an increase in serious or treatment‐related AEs, and the data were overall consistent with those of post‐marketing trials and clinical experience.^39^ Since NSIST is allowed in the tocilizumab group, the risk of infection should be a concern. However, no AEs of infection were reported in our study, because the steroid tapering was allowed for patients when symptoms improved. In addition, 40% (8/20) patients in the tocilizumab group did not use NSIST due to prior treatment failure or intolerance to side effects. Notably, three of four young or child‐bearing period female patients had irregular menstruation, and we found they took tacrolimus that is known to cause menstrual irregularities. Their menstrual cycles returned to normal when they ceased tacrolimus. It seems that menstrual irregularities might be attributed to the use of tacrolimus. Due to the short observation period and small sample size, the exact cause of the menstrual irregularities cannot be confirmed and the risk of tocilizumab in MG may be underestimated. A study with larger sample size will be needed to study the safe profile of tocilizumab.

In the era of innovative drugs, physicians should know the cost/effectiveness ratio for disease treatment and their availability. Economic aspects are also important sources of high disease burden.^2^ For eculizumab, with an estimated annual cost of $653,100, the incremental cost‐effectiveness ratio was $5,210,000 per QALY/evLYG. Efgartigimod has an incremental cost per QALY/evLYG of $2,076,000.^2^ The total cost of tocilizumab per patient‐year was $8000–11,000. Additionally, its high availability promises the sustainability of tocilizumab in clinical practice, particularly in the developing countries.

This study had several limitations. First, the sample size was relatively small, which may introduce selection and measurement bias, potentially undermine the validity and reliability of the overall representation. The measurement and random errors may compromise the statistical accuracy of the results, thus limit the external validity of the outcomes to a larger population. Although the repeated‐measures design indicated that the sample size was adequate to provide statistical power to confirm group differences, larger sample sizes was still warranted to provide more accurate information. Second, the observational design might introduce confounding bias and selection bias despite the relatively balanced baseline characteristics. Thus, we performed several sensitivity analyses to further evaluate the impact of potential confounders on the stability of the primary outcome, which were consistent with the main analyses. However, it is still impossible to eliminate unincorporated confounding factors that may result in bias. This issue will be addressed in an ongoing multicenter randomized controlled trial (NCT05067348). In addition, we further expand the cohort size and conduct long‐term observation of the existing cohort.

## 5 CONCLUSION

In conclusion, tocilizumab is safe and effective in improving the MG‐ADL score and reducing prednisone dose in refractory patients with AChR‐Ab+ gMG. Refractory MG is still a challenge for both patients and neurologists. Based on the above results, tocilizumab has the potential to be a valuable therapeutic option for such patients. The ongoing controlled randomized trial and the extension study of tocilizumab will provide additional insight on the effectiveness and long‐term safety of tocilizumab in refractory AChR‐Ab+ gMG.

## AUTHOR CONTRIBUTIONS

Dr Ting Chang had full access to all of the data in the study and takes responsibility for the integrity of the data and the accuracy of the data analysis. Zhe Ruan, Yong‐Lan Tang and Ting Gao contributed equally to this work. Concept and design: Ting Chang and Zhe Ruan. Acquisition, analysis, or interpretation of data: Yong‐Lan Tang, Ting Gao, Chun‐Hong Li, Rong‐Jing Guo, Chao Sun, Xiao‐Xi Huang. Drafting of the manuscript: Ting Chang and Zhe Ruan. Critical revision of the manuscript for important intellectual content: Ting Chang, Zhe Ruan, and Zhu‐Yi Li. Statistical analysis: Zhe Ruan and Yong‐Lan Tang. Obtained funding: Ting Chang. Administrative, technical, or material support: Rong‐Jing Guo. Supervision: Ting Chang and Zhu‐Yi Li.

## FUNDING INFORMATION

This work was supported by the discipline innovation and development plan of Tangdu hospital‐major program of clinical research (Grant no. 2021LCYJ002), Key R & D plan of Shaanxi Province (Grant no. 2021ZDLSF02‐01), the National Key Research and Development Program (Grant no. 2022YFC3501304), and the National Natural Science Foundation of China (Grant no. 82271378).

## CONFLICT OF INTEREST STATEMENT

All authors declare that are no competing interests.

## Supporting information


Data S1.

## Data Availability

The datasets, which include individual participant data and a data dictionary defining each field in the set used or analyzed during the current study, will be available upon reasonable request. Requests for data should be submitted email to changting1981@163.com or ruanzhe573291596@126.com. The data that will be made available comprise deidentified participant data. *R* syntax will be made available upon request.

## References

[1] Gilhus NE , Tzartos S , Evoli A , Palace J , Burns TM , Verschuuren J . Myasthenia gravis. Nat Rev Dis Primers. 2019;5(1):30. 31048702 10.1038/s41572-019-0079-y

[2] Lehnerer S , Jacobi J , Schilling R , et al. Burden of disease in myasthenia gravis: taking the patient's perspective. J Neurol. 2022;269(6):3050‐3063. 34800167 10.1007/s00415-021-10891-1 PMC9120127

[3] Bril V , Benatar M , Andersen H , et al. Efficacy and safety of rozanolixizumab in moderate to severe generalized myasthenia gravis: a phase 2 randomized control trial. Neurology. 2021;96(6):e853‐e865. 33219142 10.1212/WNL.0000000000011108 PMC8105899

[4] Bril V , Drużdż A , Grosskreutz J , et al. Safety and efficacy of rozanolixizumab in patients with generalised myasthenia gravis (MycarinG): a randomised, double‐blind, placebo‐controlled, adaptive phase 3 study. Lancet Neurol. 2023;22(5):383‐394. 37059507 10.1016/S1474-4422(23)00077-7

[5] Howard JF Jr , Bresch S , Genge A , et al. Safety and efficacy of zilucoplan in patients with generalised myasthenia gravis (RAISE): a randomised, double‐blind, placebo‐controlled, phase 3 study. Lancet Neurol. 2023;22(5):395‐406. 37059508 10.1016/S1474-4422(23)00080-7

[6] Howard JF Jr , Bril V , Vu T , et al. Safety, efficacy, and tolerability of efgartigimod in patients with generalised myasthenia gravis (ADAPT): a multicentre, randomised, placebo‐controlled, phase 3 trial. Lancet Neurol. 2021;20(7):526‐536. 34146511 10.1016/S1474-4422(21)00159-9

[7] Howard JF Jr , Nowak RJ , Wolfe GI , et al. Clinical effects of the self‐administered subcutaneous complement inhibitor Zilucoplan in patients with moderate to severe generalized myasthenia gravis: results of a phase 2 randomized, double‐blind, placebo‐controlled, multicenter clinical trial. JAMA Neurol. 2020;77(5):582‐592. 32065623 10.1001/jamaneurol.2019.5125 PMC7042797

[8] Howard JF Jr , Utsugisawa K , Benatar M , et al. Safety and efficacy of eculizumab in anti‐acetylcholine receptor antibody‐positive refractory generalised myasthenia gravis (REGAIN): a phase 3, randomised, double‐blind, placebo‐controlled, multicentre study. Lancet Neurol. 2017;16(12):976‐986. 29066163 10.1016/S1474-4422(17)30369-1

[9] Aricha R , Mizrachi K , Fuchs S , Souroujon MC . Blocking of IL‐6 suppresses experimental autoimmune myasthenia gravis. J Autoimmun. 2011;36(2):135‐141. 21193288 10.1016/j.jaut.2010.12.001

[10] Kimura A , Kishimoto T . IL‐6: regulator of Treg/Th17 balance. Eur J Immunol. 2010;40(7):1830‐1835. 20583029 10.1002/eji.201040391

[11] Zhang C , Zhang M , Qiu W , et al. Safety and efficacy of tocilizumab versus azathioprine in highly relapsing neuromyelitis optica spectrum disorder (TANGO): an open‐label, multicentre, randomised, phase 2 trial. Lancet Neurol. 2020;19(5):391‐401. 32333897 10.1016/S1474-4422(20)30070-3 PMC7935423

[12] Smolen JS , Beaulieu A , Rubbert‐Roth A , et al. Effect of interleukin‐6 receptor inhibition with tocilizumab in patients with rheumatoid arthritis (OPTION study): a double‐blind, placebo‐controlled, randomised trial. Lancet. 2008;371(9617):987‐997. 18358926 10.1016/S0140-6736(08)60453-5

[13] Jonsson DI , Pirskanen R , Piehl F . Beneficial effect of tocilizumab in myasthenia gravis refractory to rituximab. Neuromuscul Disord. 2017;27(6):565‐568. 28433474 10.1016/j.nmd.2017.03.007

[14] Sanders DB , Wolfe GI , Benatar M , et al. International consensus guidance for management of myasthenia gravis: executive summary. Neurology. 2016;87(4):419‐425. 27358333 10.1212/WNL.0000000000002790 PMC4977114

[15] Li Z , Zhang C , Chang T , et al. A multicentre, prospective, double‐blind study comparing the accuracy of autoantibody diagnostic assays in myasthenia gravis: the SCREAM study. Lancet Reg Health West Pac. 2023;38:100846. 37554174 10.1016/j.lanwpc.2023.100846 PMC10404541

[16] Muppidi S , Silvestri NJ , Tan R , Riggs K , Leighton T , Phillips GA . Utilization of MG‐ADL in myasthenia gravis clinical research and care. Muscle Nerve. 2022;65(6):630‐639. 34989427 10.1002/mus.27476 PMC9302997

[17] Barnett C , Katzberg H , Nabavi M , Bril V . The quantitative myasthenia gravis score: comparison with clinical, electrophysiological, and laboratory markers. J Clin Neuromuscul Dis. 2012;13(4):201‐205. 22622164 10.1097/CND.0b013e31824619d5

[18] Burns TM , Conaway M , Sanders DB . The MG composite: a valid and reliable outcome measure for myasthenia gravis. Neurology. 2010;74(18):1434‐1440. 20439845 10.1212/WNL.0b013e3181dc1b1e PMC3462556

[19] Brown H , Prescott R . Applied Mixed Models in Medicine. John Wiley & Sons; 2006.

[20] Diggle PJ , Liang KY , Zeger SL . Analysis of Longitudinal Data. Oxford University Press; 1994.

[21] Salazar A , Ojeda B , Dueñas M , Fernández F , Failde I . Simple generalized estimating equations (GEEs) and weighted generalized estimating equations (WGEEs) in longitudinal studies with dropouts: guidelines and implementation in R. Stat Med. 2016;35(19):3424‐3448. 27059703 10.1002/sim.6947

[22] Dresser L , Wlodarski R , Rezania K , Soliven B . Myasthenia gravis: epidemiology, pathophysiology and clinical manifestations. J Clin Med. 2021;10(11):2235. 34064035 10.3390/jcm10112235 PMC8196750

[23] Keung B , Robeson KR , DiCapua DB , et al. Long‐term benefit of rituximab in MuSK autoantibody myasthenia gravis patients. J Neurol Neurosurg Psychiatry. 2013;84(12):1407‐1409. 23761915 10.1136/jnnp-2012-303664

[24] Molimard A , Gitiaux C , Barnerias C , et al. Rituximab therapy in the treatment of juvenile myasthenia gravis: the French experience. Neurology. 2022;98(23):e2368‐e2376. 35314497 10.1212/WNL.0000000000200288

[25] Silvestri NJ , Wolfe GI . Rituximab in treatment‐refractory myasthenia gravis. JAMA Neurol. 2017;74(1):21‐23. 27892981 10.1001/jamaneurol.2016.4367

[26] Singh N , Goyal V . Rituximab as induction therapy in refractory myasthenia gravis: 18 month follow‐up study. J Neurol. 2019;266(7):1596‐1600. 30919039 10.1007/s00415-019-09296-y

[27] Topakian R , Zimprich F , Iglseder S , et al. High efficacy of rituximab for myasthenia gravis: a comprehensive nationwide study in Austria. J Neurol. 2019;266(3):699‐706. 30649616 10.1007/s00415-019-09191-6

[28] Zhou Y , Yan C , Gu X , et al. Short‐term effect of low‐dose rituximab on myasthenia gravis with muscle‐specific tyrosine kinase antibody. Muscle Nerve. 2021;63(6):824‐830. 33745138 10.1002/mus.27233

[29] Piehl F , Eriksson‐Dufva A , Budzianowska A , et al. Efficacy and safety of rituximab for new‐onset generalized myasthenia gravis: the RINOMAX randomized clinical trial. JAMA Neurol. 2022;79(11):1105‐1112. 36121672 10.1001/jamaneurol.2022.2887 PMC9486640

[30] Nurieva RI , Chung Y , Martinez GJ , et al. Bcl6 mediates the development of T follicular helper cells. Science. 2009;325(5943):1001‐1005. 19628815 10.1126/science.1176676 PMC2857334

[31] Xiao F , Han M , Rui K , et al. New insights into follicular helper T cell response and regulation in autoimmune pathogenesis. Cell Mol Immunol. 2021;18(6):1610‐1612. 33972739 10.1038/s41423-021-00688-7 PMC8166848

[32] Das S , Vital EM , Horton S , et al. Abatacept or tocilizumab after rituximab in rheumatoid arthritis? An exploratory study suggests non‐response to rituximab is associated with persistently high IL‐6 and better clinical response to IL‐6 blocking therapy. Ann Rheum Dis. 2014;73(5):909‐912. 24385201 10.1136/annrheumdis-2013-204417

[33] Traboulsee A , Greenberg BM , Bennett JL , et al. Safety and efficacy of satralizumab monotherapy in neuromyelitis optica spectrum disorder: a randomised, double‐blind, multicentre, placebo‐controlled phase 3 trial. Lancet Neurol. 2020;19(5):402‐412. 32333898 10.1016/S1474-4422(20)30078-8 PMC7935419

[34] Jia D , Zhang F , Li H , et al. Responsiveness to Tocilizumab in anti‐acetylcholine receptor‐positive generalized myasthenia gravis. Aging Dis. 2023;15:824‐830. 10.14336/AD.2023.0528 PMC10917550 37450932

[35] Filippi M , Bar‐Or A , Piehl F , et al. Multiple sclerosis. Nat Rev Dis Primers. 2018;4(1):43. 30410033 10.1038/s41572-018-0041-4

[36] Saki A , Rajaei E , Rahim F . Safety and efficacy of tocilizumab for rheumatoid arthritis: a systematic review and meta‐analysis of clinical trial studies. Reumatologia. 2021;59(3):169‐179. 34538944 10.5114/reum.2021.107026 PMC8436803

[37] Jones G , Panova E . New insights and long‐term safety of tocilizumab in rheumatoid arthritis. Ther Adv Musculoskelet dis. 2018;10(10):195‐199. 30327685 10.1177/1759720X18798462 PMC6178374

[38] Bykerk VP , Östör AJK , Alvaro‐Gracia J , et al. Long‐term safety and effectiveness of tocilizumab in patients with rheumatoid arthritis and inadequate responses to csDMARDs and/or TNF inhibitors: an open‐label study close to clinical practice. Clin Rheumatol. 2019;38(9):2411‐2421. 31028551 10.1007/s10067-019-04535-z

[39] Scott LJ . Tocilizumab: a review in rheumatoid arthritis. Drugs. 2017;77(17):1865‐1879. 29094311 10.1007/s40265-017-0829-7 PMC5736769

