# Supplementary material for: Efficacy and safety of tocilizumab in patients with refractory generalized myasthenia gravis
Source: CNS Neurosci Ther. 2024 Jun 18;30(6):e14793. doi: 10.1111/cns.14793 (PMC11187874; doi:10.1111/cns.14793)
Supplement: Supplementary file 1 — Data S1. [file CNS-30-e14793-s001.zip › research proposal.docx]

**Efficacy and safety of tocilizumab in the treatment of generalizaed myasthenia gravis: a single-center, prospective cohort study**

| Registration Number: | ChiCTR2100043273 |
| --- | --- |
| Date of Last Refreshed on: | 2021-05-28 |
| Date of Registration: | 2021-02-09 |
| Registration Status: | Prospective Registration |
| Public Title: | Efficacy and safety of tocilizumab in the treatment of generalizaed myasthenia gravis: a single-center, prospective cohort study |
| English Acronym: |  |
| Scientific Title: | Efficacy and safety of tocilizumab in the treatment of generalizaed myasthenia gravis: a single-center, prospective cohort study |
| Study Subject ID: |  |
| The Registration Number of the Partner Registry or Other Register: |  |
| Applicant: | Ruan Zhe |
| Applicant Telephone: | +86 15829290162 |
| Applicant Fax: |  |
| Applicant E-mail: | ruanzhe573291596@126.com |
| Applicant Address: | 569 Xinsi Road, Baqiao District, Xi'an, Shaanxi, China |
| Applicant's Institution: | The Second Affiliated Hospital of Air Force Medical University |
| Study Leader: | Chang Ting |
| Study leader's Telephone: | +86 29-84778845 |
| Study Leader's Fax: |  |
| Study Leader's E-mail: | changting1981@163.com |
| Study Leader's Address: | 569 Xinsi Road, Baqiao District, Xi'an, Shaanxi, China |
| Affiliation of the Leader: | The Second Affiliated Hospital of Air Force Medical University |
| Approved by Ethic Committee: | Yes |
| Approved No. of Ethic Committee: | No. 202102-06 |
| Name of the Ethic Committee: | Medical Ethics Committee of Tangdu Hospital of the Fourth Military Medical University |
| Date of Approved by Ethic Committee: | 2021-02-01 |
| Contact Name of the Ethic Committee: | Li Shicao |
| Contact Address of the Ethic Committee: | Room 304, Clinical Teaching Building, The Second Affiliated Hospital of Air Force Medical University, 569 Xinsi Road, Baqiao District, Xi’an, Shaanxi, China |
| Contact Phone of the Ethic Committee: | +86 29-84777631 |
| Contact Email of the Ethic Committee | tangduec@126.com |
| Primary Sponsor: | Department of Neurology, The Second Affiliated Hospital of Air Force Medical University |
| Primary Sponsor's Address: | 569 Xinsi Road, Baqiao District, Xi’an, Shaanxi, China |
| Secondary Sponsor: | Country: China |
|  | Province: Shaanxi |
|  | City: Xi'an |
|  | Institution Hospital: The Second Affiliated Hospital of Air Force Medical University |
|  | Address: 569 Xinsi Road, Baqiao District |
| Source(s) of Funding: | Top Talent Fund, The Second Affiliated Hospital of Air Force Medical University |
|  | National Natural Science Foundation of China (81671233) |
| Target Disease: | Myasthenia Gravis |
| Study Type: | Observational Study |
| Study Phase: | N/A |
| Study Design: | Non randomized Control |
| Objectives of Study: | 1. To evaluate the effectiveness of tocilizumab in the treatment of generalizaed AChR-Ab (+) MG. |
|  | 2. Compare the effectiveness of tocilizumab and conventional immunotherapy. |
| Description for Medicine or Protocol of Treatment in Detail: |  |
| Inclusion Criteria: | 1. At the research center, the neurologist was diagnosed with myasthenia gravis according to the diagnostic criteria of the 2015 Chinese Myasthenia Gravis Guidelines. |
|  | 2. Aged ≥ 18 years. |
|  | 3. MGFA classification IIa-IVa, acetylcholine receptor antibody (+). |
|  | 4. Receiving traditional immunotherapy and the drug dose is stable for more than 3 month before the first evaluation of the group; or after the physician's evaluation, it is determined that the tocilizumab combined hormone therapy program will be accepted. |
|  | 5. Agree to accept blood sample collection. |
|  | 6. Voluntarily sign the informed consent form. |
| Exclusion Criteria: | 1. Complicated with other neurological diseases, which interferes with the assessment of MG status. |
|  | 2. Combined with unresected thymoma. |
|  | 3. Pregnant or lactating women. |
|  | 4. Women of childbearing age who have family planning. |
|  | 5. Severe anxiety, depression and schizophrenia. |
|  | 6. Suffer from other serious systemic diseases, and the expected survival period is less than 6 months. |
|  | 7. Patients considered by the researcher to be unsuitable to participate in the study. |
| Study Execute Time: | From 2021-02-01 To 2021-10-31 |
| Recruiting Time: | From 2021-02-20 To 2021-10-31 |
| Interventions: |  |
|  | Tocilizumab Treatment Group |
|  | Intervention: Tocilizumab + Oral Immunosuppressant |
|  | Conventional Immunotherapy Group |
|  | Intervention: Oral Immunosuppressant |
| Countries of Recruitment and Research Settings: | Country: China |
|  | Province: Shaanxi |
|  | City: Xi'an |
|  | Institution Hospital: The Second Affiliated Hospital of Air Force Medical University |
|  | Level of the Institution: Tertiary A |
| Outcomes: | Change in MG-ADL score from baseline at study week 24. |
| Outcome: | Proportion of patients with symptom relief at study week 24 (MG-ADL improvement ≥ 2 points). |
| Outcome: | Change in QMG score from baseline at study week 12. |
| Outcome: | Proportion of patients with symptom relief in the 12th week of the study (MG-ADL improvement ≥ 2 points). |
| Outcome: | The proportion of patients whose hormones were reduced to less than 10 mg/d at week 12 of the study. |
| Outcome: | The average daily dose of steroids at the 12th week of the study. |
| Outcome: | The change in QoL-15 score from baseline at week 12 of the study. |
| Outcome: | The change in QMG score from baseline at week 24 of the study. |
| Outcome: | Proportion of patients with symptom relief in the 24th week of the study (MG-ADL improvement ≥ 2 points). |
| Outcome: | The proportion of patients whose hormones were reduced to less than 10 mg/d at week 24 of the study. |
| Outcome: | The average daily dose of steroids at week 24 of the study. |
| Outcome: | The change in QoL-15 score from baseline at the 24th week of the study. |
| Recruiting Status: | Recruiting |
| Participant Age: | Min Age 18 Years, Max Age 80 Years |
| Gender: | Both |
| Randomization Procedure: | Non randomization |
| Calculated Results After the Study Completed Public Access: | Private |
| Blinding: | Open-label |
| IPD Sharing: | No |
| The Way of Sharing IPD: | Paper Submission |
| Data Collection and Management: | ResMan |
| Data and Safety Monitoring Committee: | Yes |
