# Supplementary material for: Efficacy and safety of tocilizumab in patients with refractory generalized myasthenia gravis
Source: CNS Neurosci Ther. 2024 Jun 18;30(6):e14793. doi: 10.1111/cns.14793 (PMC11187874; doi:10.1111/cns.14793)
Supplement: Supplementary file 1 — Data S1. [file CNS-30-e14793-s001.zip › Supplements.docx]

**eTable 1. R packages used in statistical analyses**

**eTable 2. Individual immunotherapy courses of included 34 patients**

**eTable 3. Adjusted mean of all scores measures and the daily prednisone dose from week 4 at week 24 in two groups**

**eTable 4. The proportions of MG­ADL and QMG responders from week 4 at week 24 in two groups**

**eTable 5. Sensitivity analysis for MG-ADL score by generalized estimated equation**

**eTable 6. Sensitivity analysis for QMG score by generalized estimated equation**

**eTable 7. Sensitivity analysis for MGC score by generalized estimated equation**

**eTable 8. Sensitivity analysis for daily prednisone dose by generalized estimated equation**

**eTable 9. Details of adverse events occurring in the tocilizumab group**

**eFigure 1. Individual change of included 34 patients in the MG-ADL, QMG, and MGC scores and daily prednisone dose from baseline to 24 weeks**

**eFigure 2. Adjusted mean of MG-ADL (A), QMG (B), MGC (C) scores and daily prednisone dose changes from baseline to 24 weeks**

**eTable 1. R packages used in statistical analyses**

| **R package** | **Version** |
| --- | --- |
| geepack | 1.3.9 |
| emmeans | 1.8.2 |
| dplyr | 1.0.10 |
| ggplot2 | 3.3.6 |
| tableone | 0.13.2 |

**eTable2. Individual immunotherapy courses of included 34 patients**

| **Number** | **Group** | **Sex** | **Age** | **Immunotherapies** |
| --- | --- | --- | --- | --- |
| 1 | Tocilizumab | female | 39 | inadequate response to steroids and TAC |
| 2 | Tocilizumab | male | 76 | inadequate response to steroids and TAC; steroids tapering-induced MC |
| 3 | Tocilizumab | male | 74 | inadequate response to steroids and TAC |
| 4 | Tocilizumab | male | 58 | inadequate response to steroids and AZA; hard-to-control hyperglycemia |
| 5 | Tocilizumab | female | 18 | combination therapy with steroids and TAC failed to achieve the treatment goal, steroids tapering-induced recurrent relapses |
| 6 | Tocilizumab | male | 64 | inadequate response to steroids and MMF; steroids tapering-induced relapses |
| 7 | Tocilizumab | female | 24 | intolerance to AZA (exfoliative dermatitis); inadequate response to steroids and TAC; MC occurred during high-dose steroids maintenance |
| 8 | Tocilizumab | male | 84 | inadequate response to steroids and AZA, TAC, MMF; recurrent bulbar symptoms |
| 9 | Tocilizumab | female | 57 | inadequate response to steroids and MMF; comorbid rheumatoid arthritis |
| 10 | Tocilizumab | female | 65 | comorbid osteoporosis and diabetes; intolerance to steroids and TAC (ketosis) |
| 11 | Tocilizumab | female | 19 | inadequate response to MMF and TAC, recurrent relapse, myasthenic symptoms fluctuate with the menstrual cycle |
| 12 | Tocilizumab | female | 68 | intolerance to steroids (osteonecrosis of the femoral head), inadequate response to AZA |
| 13 | Tocilizumab | male | 28 | inadequate response to steroids and TAC; ocular fixation for seven years |
| 14 | Tocilizumab | female | 55 | comorbid paroxysmal atrial fibrillation and cervical intraepithelial neoplasia; steroids and AZA tapering-induced recurrent relapses |
| 15 | Tocilizumab | female | 69 | steroids tapering-induced recurrent relapses; comorbid lower-limb venous thrombosis; intolerance to steroids (obesity) |
| 16 | Tocilizumab | male | 57 | inadequate response to steroids and TAC |
| 17 | Tocilizumab | female | 59 | steroids tapering-induced MC; inadequate response to IVIg and RTX |
| 18 | Tocilizumab | female | 18 | inadequate response to MMF and RTX; myasthenic symptoms fluctuate with the menstrual cycle |
| 19 | Tocilizumab | female | 86 | advanced age; comorbid hypertension, coronary heart disease and chronic cardiac dysfunction |
| 20 | Tocilizumab | male | 57 | inadequate response to steroids; history of erosive gastritis |
| 21 | Control | female | 54 | inadequate response to AZA and RTX; recurrent relapses |
| 22 | Control | female | 66 | inadequate response to steroids, TAC and pyridostigmine |
| 23 | Control | male | 45 | inadequate response to AZA and TAC; steroids tapering-induced recurrent relapses |
| 24 | Control | male | 61 | inadequate response to AZA; mild improvement after receiving once 500mg RTX |
| 25 | Control | male | 37 | inadequate response to steroids and TAC |
| 26 | Control | male | 64 | inadequate response to steroids and MMF |
| 27 | Control | male | 57 | inadequate response to MMF |
| 28 | Control | female | 25 | inadequate response to CA and TAC |

**eTable2. Individual immunotherapy courses of included 34 patients (continued)**

| 29 | Control | female | 24 | inadequate response to steroids and TAC; recurrent relapses four times |
| --- | --- | --- | --- | --- |
| 30 | Control | female | 26 | inadequate response to steroids and TAC; steroids tapering-induced recurrent relapses |
| 31 | Control | female | 23 | inadequate response to steroids and AZA |
| 32 | Control | female | 28 | inadequate response to steroids and AZA; pregnancy demand |
| 33 | Control | female | 71 | advanced age; comorbid hypertension, coronary heart disease and diabetes; inadequate response to AZA |
| 34 | Control | male | 60 | inadequate response to MMF; severe pulmonary infection after receiving once 500mg RTX |

**Abbreviations:** TAC, Tacrolimus; MC, Myasthenic crisis; AZA, Azathioprine; MMF, Mycophenolate mofetil; RTX, Rituximab; CA, Cyclosporine A.

| **Outcomes** | **Control**  **(n=14)** | **Tocilizumab**  **(n=20)** |
| --- | --- | --- |
|  |  |  |
| **MG-ADL score, adjusted mean (95 CI%) ^a^** | | |
| week 4 | 8.0 (7.0 to 9.0) | 4.7 (3.7 to 5.6) |
| week 8 | 7.7 (6.9 to 8.6) | 3.3 (2.4 to 4.2) |
| week 12 | 7.5 (6.4 to 8.7) | 2.5 (1.7 to 3.3) |
| week 16 | 6.7 (5.8 to 7.7) | 2.3 (1.5 to 3.0) |
| week 20 | 6.6 (5.6 to 7.6) | 2.2 (1.2 to 3.3) |
| week 24 | 6.7 (5.4 to 8.0) | 2.2 (0.9 to 3.5) |
| **QMG score, adjusted mean (95 CI%) ^b^** | | |
| week 4 | 14.7 (13.0 to 16.4) | 11.9 (10.3 to 13.4) |
| week 8 | 15.1 (13.8 to 16.4) | 10.5 (8.8 to 12.2) |
| week 12 | 14.6 (13.3 to 16.0) | 9.4 (7.8 to 11.0) |
| week 16 | 13.6 (12.5 to 14.7) | 8.3 (6.9 to 9.7) |
| week 20 | 14.1 (12.0 to 16.2) | 8.7 (6.6 to 10.8) |
| week 24 | 13.9 (11.5 to 16.3) | 8.1 (5.9 to 10.5) |
| **MGC score, adjusted mean (95 CI%) ^c^** | | |
| week 4 | 16.6 (14.0 to 19.1) | 8.7 (6.5 to 10.8) |
| week 8 | 15.0 (12.5 to 17.6) | 6.6 (4.6 to 8.5) |
| week 12 | 14.9 (12.4 to 17.3) | 4.8 (3.1 to 6.6) |
| week 16 | 14.1 (11.9 to 16.2) | 3.9 (2.0 to 5.7) |
| week 20 | 13.2 (10.9 to 15.5) | 4.0 (1.6 to 6.4) |
| week 24 | 12.9 (10.2 to 15.7) | 5.0 (1.5 to 8.5) |
| **Daily prednisone dose, adjusted mean (95 CI%), mg ^d^** | | |
| week 4 | 29.3 (26.3 to 32.3) | 22.5 (19.6 to 25.4) |
| week 8 | 29.0 (25.0 to 32.9) | 19.7 (16.6 to 22.9) |
| week 12 | 29.3 (24.9 to 33.8) | 17.0 (13.9 to 20.1) |
| week 16 | 27.9 (24.0 to 31.8) | 14.6 (10.9 to 18.3) |
| week 20 | 26.8 (22.7 to 30.9) | 12.0 (7.8 to 16.1) |
| week 24 | 24.8 (20.8 to 28.8) | 11.7 (6.8 to 16.7) |

**eTable 3.** **Adjusted mean of all scores measures and the daily prednisone dose from week 4 at week 24 in two groups**

**Abbreviations:** MG-ADL, Myasthenia Gravis Activities of Daily Living; QMG, Quantitative Myasthenia Gravis; MGC, Myasthenia Gravis Composite

^a^ Generalized estimated equation model adjusting for baseline MG-ADL score.

^b^ Generalized estimated equation model adjusting for baseline QMG score.

^c^ Generalized estimated equation model adjusting for baseline MGC score.

^d^ Generalized estimated equation model adjusting for baseline daily prednisone dose.

**eTable 4: The proportions of MG­ADL and QMG responders from week 4 at week 24 in two groups**

| **Outcomes** | **Control**  **(n=14)** | | **Tocilizumab**  **(n=20)** | | ***P*-value ^b^** |
| --- | --- | --- | --- | --- | --- |
|  | **No. (%)** | **95% CI for the proportion of outcome, % ^a^** | **No. (%)** | **95% CI for the proportion of outcome ^a^** |  |
| **MG-ADL responders** | | | | | |
| week 4 | 1 (7.1) | 0.2 - 33.9 | 18 (90.0) | 68.3 - 98.8 | < .001 |
| week 8 | 1 (7.1) | 0.2 - 33.9 | 18 (90.0) | 68.3 - 98.8 | < .001 |
| week 12 | 4 (28.6) | 8.4 - 58.1 | 19 (95.0) | 75.1- 99.9 | < .001 |
| week 16 | 9 (64.3) | 35.1 - 87.2 | 19 (95.0) | 75.1- 99.9 | .06 |
| week 20 | 7 (50.0) | 23.0 - 77.0 | 19 (95.0) | 75.1- 99.9 | .004 |
| week 24 | 5 (35.7) | 12.8 - 64.9 | 19 (95.0) | 75.1- 99.9 | < .001 |
| **QMG responders** | | | | | |
| week 4 | 2 (16.7) | 2.0 - 48.4 | 15 (75.0) | 50.9 - 91.3 | .002 |
| week 8 | 2 (15.4) | 1.9 - 45.4 | 17 (85.0) | 62.1 - 96.8 | < .001 |
| week 12 | 4 (28.6) | 8.3 - 58.1 | 18 (90.0) | 68.3 - 98.8 | < .001 |
| week 16 | 5 (38.5) | 13.9 - 68.4 | 19 (95.0) | 75.1 - 99.9 | < .001 |
| week 20 | 5 (41.7) | 15.2 - 72.3 | 18 (90.0) | 68.3 - 98.8 | .006 |
| week 24 | 6 (42.9) | 17.7 – 71.1 | 18 (90.0) | 68.3 - 98.8 | .006 |

^a^ Calculated by binomial test

^b^ Fisher’s exact test

**eTable 5. Sensitivity analysis for MG-ADL score by generalized estimated equation**

| **MG-ADL** | **Tocilizumab vs. Control** | | **Group-by-Time Interaction effect** | **Group effect** | **Time effect** |
| --- | --- | --- | --- | --- | --- |
|  | **Adjusted Mean Difference (95% CI) ^a^** | ***P* value** |  |  |  |
| **Adjustment for all variables** | | |  |  |  |
| Baseline | - | - | .01 | <.001 | <.001 |
| week 4 | -3.5 (-4.7 to -2.0) | <.001 |  |  |  |
| week 8 | -4.6 (-5.7 to -3.2) | <.001 |  |  |  |
| week 12 | -5.1 (-6.4 to -3.6) | <.001 |  |  |  |
| week 16 | -4.6 (-5.7 to -3.2) | <.001 |  |  |  |
| week 20 | -4.5 (-5.9 to -2.9) | <.001 |  |  |  |
| week 24 | -4.6 (-6.4 to -2.6) | <.001 |  |  |  |
| **Adjustment for baseline MG-ADL score, sex and thymic status ^a^** | | |  |  |  |
| Baseline | - | - | .01 | <.001 | <.001 |
| week 4 | -3.6 (-4.7 to -2.0) | <.001 |  |  |  |
| week 8 | -4.7 (-5.7 to -3.2) | <.001 |  |  |  |
| week 12 | -5.3 (-6.4 to -3.6) | <.001 |  |  |  |
| week 16 | -4.7 (-5.7 to -3.2) | <.001 |  |  |  |
| week 20 | -4.6 (-5.9 to -2.9) | <.001 |  |  |  |
| week 24 | -4.7 (-6.4 to -2.6) | <.001 |  |  |  |
| **Adjustment for baseline MG-ADL score** | | |  |  |  |
| Baseline | - | - | .01 | <.001 | <.001 |
| week 4 | -3.4 (-4.7 to -2.0) | <.001 |  |  |  |
| week 8 | -4.5 (-5.7 to -3.2) | <.001 |  |  |  |
| week 12 | -5.0 (-6.4 to -3.6) | <.001 |  |  |  |
| week 16 | -4.5 (-5.7 to -3.2) | <.001 |  |  |  |
| week 20 | -4.4 (-5.9 to -2.9) | <.001 |  |  |  |
| week 24 | -4.5 (-6.4 to -2.6) | <.001 |  |  |  |

**Abbreviations:** MG-ADL, Myasthenia Gravis Activities of Daily Living

^a^ Sex and thymic status were adjusted as covariates due to they had significant fixed effects in the full model.

**eTable 6. Sensitivity analysis for QMG score by generalized estimated equation**

| **QMG score** | **Tocilizumab vs. Control** | | **Group-by-Time Interaction effect** | **Group effect** | **Time effect** |
| --- | --- | --- | --- | --- | --- |
|  | **Adjusted Mean Difference (95% CI) ^a^** | ***P* value** |  |  |  |
| **Adjustment for all variables** | | |  |  |  |
| Baseline | - | - | .20 | .008 | <.001 |
| week 4 | -2.9 (-5.1 to -0.6) | .04 |  |  |  |
| week 8 | -4.3 (-6.8 to -2.4) | < .001 |  |  |  |
| week 12 | -5.1 (-7.4 to -3.0) | < .001 |  |  |  |
| week 16 | -5.0 (-7.1 to -3.4) | < .001 |  |  |  |
| week 20 | -5.2 (-8.5 to -2.4) | < .001 |  |  |  |
| week 24 | -5.6 (-9.2 to -2.4) | < .001 |  |  |  |
| **Adjustment for baseline QMG score、sex and thymic status ^a^** | | |  |  |  |
| Baseline | - | - | .15 | .008 | <.001 |
| week 4 | -3.1 (-5.1 to -0.6) | .03 |  |  |  |
| week 8 | -4.6 (-6.8 to -2.4) | <.001 |  |  |  |
| week 12 | -5.3 (-7.4 to -3.0) | <.001 |  |  |  |
| week 16 | -5.4 (-7.1 to -3.4) | <.001 |  |  |  |
| week 20 | -5.5 (-8.5 to -2.4) | < .001 |  |  |  |
| week 24 | -5.9 (-9.2 to -2.4) | < .001 |  |  |  |
| **Adjustment for baseline QMG score** | | |  |  |  |
| Baseline | - | - | .05 | .008 | <.001 |
| week 4 | -2.8 (-5.1 to -0.6) | .09 |  |  |  |
| week 8 | -4.6 (-6.8 to -2.4) | <.001 |  |  |  |
| week 12 | -5.2 (-7.4 to -3.0) | <.001 |  |  |  |
| week 16 | -5.3 (-7.1 to -3.4) | <.001 |  |  |  |
| week 20 | -5.4 (-8.5 to -2.4) | .003 |  |  |  |
| week 24 | -5.8 (-9.2 to -2.4) | .004 |  |  |  |

**Abbreviations:** QMG, Quantitative Myasthenia Gravis

^a^ Sex and thymic status were adjusted as covariates due to they had significant fixed effects in the full model.

**eTable 7. Sensitivity analysis for MGC score by generalized estimated equation**

| **MGC score** | **Tocilizumab vs. Control** | | **Group-by-Time Interaction effect** | **Group effect** | **Time effect** |
| --- | --- | --- | --- | --- | --- |
|  | **Adjusted Mean Difference (95% CI) ^a^** | ***P* value** |  |  |  |
| **Adjustment for all variables** | | |  |  |  |
| Baseline | - | - | .31 | <.001 | <.001 |
| week 4 | -7.9 (-11.2 to -4.6) | <.001 |  |  |  |
| week 8 | -8.2 (-11.7 to -5.2) | <.001 |  |  |  |
| week 12 | -9.9 (-13.1 to -7.0) | <.001 |  |  |  |
| week 16 | -9.8 (-13.0 to -7.4) | <.001 |  |  |  |
| week 20 | -9.0 (-12.5 to -5.8) | <.001 |  |  |  |
| week 24 | -7.8 (-12.5 to -3.5) | <.001 |  |  |  |
| **Adjustment for baseline MGC score、sex and thymic status ^a^** | | |  |  |  |
| Baseline | - | - | .25 | <.001 | <.001 |
| week 4 | -8.4 (-11.2 to -4.6) | <.001 |  |  |  |
| week 8 | -8.8 (-11.7 to -5.2) | <.001 |  |  |  |
| week 12 | -10.5 (-13.1 to -7.0) | <.001 |  |  |  |
| week 16 | -10.6 (-13.0 to -7.4) | <.001 |  |  |  |
| week 20 | -9.7 (-12.5 to -5.8) | <.001 |  |  |  |
| week 24 | -8.4 (-12.5 to -3.5) | <.001 |  |  |  |
| **Adjustment for baseline MGC score** | | |  |  |  |
| Baseline | - | - | .26 | <.001 | <.001 |
| week 4 | -7.9 (-11.2 to -4.6) | <.001 |  |  |  |
| week 8 | -8.5 (-11.7 to -5.2) | <.001 |  |  |  |
| week 12 | -10.1 (-13.1 to -7.0) | <.001 |  |  |  |
| week 16 | -10.2 (-13.0 to -7.4) | <.001 |  |  |  |
| week 20 | -9.2 (-12.5 to -5.8) | <.001 |  |  |  |
| week 24 | -8.0 (-12.5 to -3.5) | .003 |  |  |  |

**Abbreviations:** MGC, Myasthenia Gravis Composite

^a^ Sex and thymic status were adjusted as covariates due to they had significant fixed effects in the full model.

**eTable 8. Sensitivity analysis for daily prednisone dose by generalized estimated equation**

| **Daily prednisone dose** | **Tocilizumab vs. Control** | | **Group-by-Time Interaction effect** | **Group effect** | **Time effect** |
| --- | --- | --- | --- | --- | --- |
|  | **Adjusted Mean Difference (95% CI) ^a^** | ***P* value** |  |  |  |
| **Adjustment for all variables** | | |  |  |  |
| Baseline | - | - | .27 | <.001 | .002 |
| week 4 | -5.1 (-11.0 to -2.7) | .22 |  |  |  |
| week 8 | -7.5 (-14.3 to -4.2) | .04 |  |  |  |
| week 12 | -10.6 (-17.7 to -6.9) | .001 |  |  |  |
| week 16 | -11.6 (-18.7 to -7.9) | <.001 |  |  |  |
| week 20 | -13.1 (-20.7 to -9.0) | <.001 |  |  |  |
| week 24 | -11.4 (-19.4 to -6.7) | .002 |  |  |  |
| **Adjustment for baseline daily prednisone dose、age and thymic status ^a^** | | |  |  |  |
| Baseline | - | - | .27 | <.001 | .002 |
| week 4 | -5.2 (-11.0 to -2.7) | .17 |  |  |  |
| week 8 | -7.6 (-14.3 to -4.2) | .03 |  |  |  |
| week 12 | -10.7 (-17.7 to -6.9) | <.001 |  |  |  |
| week 16 | -11.6 (-18.7 to -7.9) | <.001 |  |  |  |
| week 20 | -13.2 (-20.7 to -9.0) | <.001 |  |  |  |
| week 24 | -11.4 (-19.4 to -6.7) | .002 |  |  |  |
| **Adjustment for baseline daily prednisone dose** | | |  |  |  |
| Baseline | - | - | .27 | <.001 | .002 |
| week 4 | -6.8 (-11.0 to -2.7) | .008 |  |  |  |
| week 8 | -9.2 (-14.3 to -4.2) | .002 |  |  |  |
| week 12 | -12.3 (-17.7 to -6.9) | <.001 |  |  |  |
| week 16 | -13.3 (-18.7 to -7.9) | <.001 |  |  |  |
| week 20 | -14.8 (-20.7 to -9.0) | <.001 |  |  |  |
| week 24 | -13.1 (-19.4 to -6.7) | <.001 |  |  |  |

^a^ Sex and thymic status covariates were adjusted because they had significant fixed effects in the full model.

**eTable 9. Details of adverse events occurring in the tocilizumab group**

| **Number** | **Sex** | **Age** | **Adverse events** | **Grade** | **Outcome** |
| --- | --- | --- | --- | --- | --- |
| 1 | female | 39 | Irregular menstruation | 1 | Normal menstruation returned after the study completion |
| 2 | male | 76 | Thrombocytopenia | 1 | Upon completion of the study, the platelet count returned to normal with no treatment given |
| 4 | male | 58 | Thrombocytopenia | 1 | Upon completion of the study, the platelet count returned to normal with no treatment given |
| 5 | female | 18 | Irregular menstruation | 2 | Normal menstruation returned after the study completion |
| 8 | female | 57 | Thrombocytopenia | 1 | Upon completion of the study, the platelet count returned to normal with no treatment given |
| 11 | female | 19 | Irregular menstruation | 1 | Normal menstruation returned after the study completion |
| 13 | male | 28 | Hyperlipidemia | 2 | Plasma lipids returned to normal after taking lipid-lowering medication |
| 17 | female | 59 | Elevated alanine aminotransferase | 1 | Alanine aminotransferase returned to normal with no treatment given |
| 17 | female | 59 | Thrombocytopenia | 1 | Upon completion of the study, the platelet count returned to normal with no treatment given |
| 18 | female | 18 | Abdominal pain | 2 | The abdominal pain disappeared after intramuscular anisodamine |

**
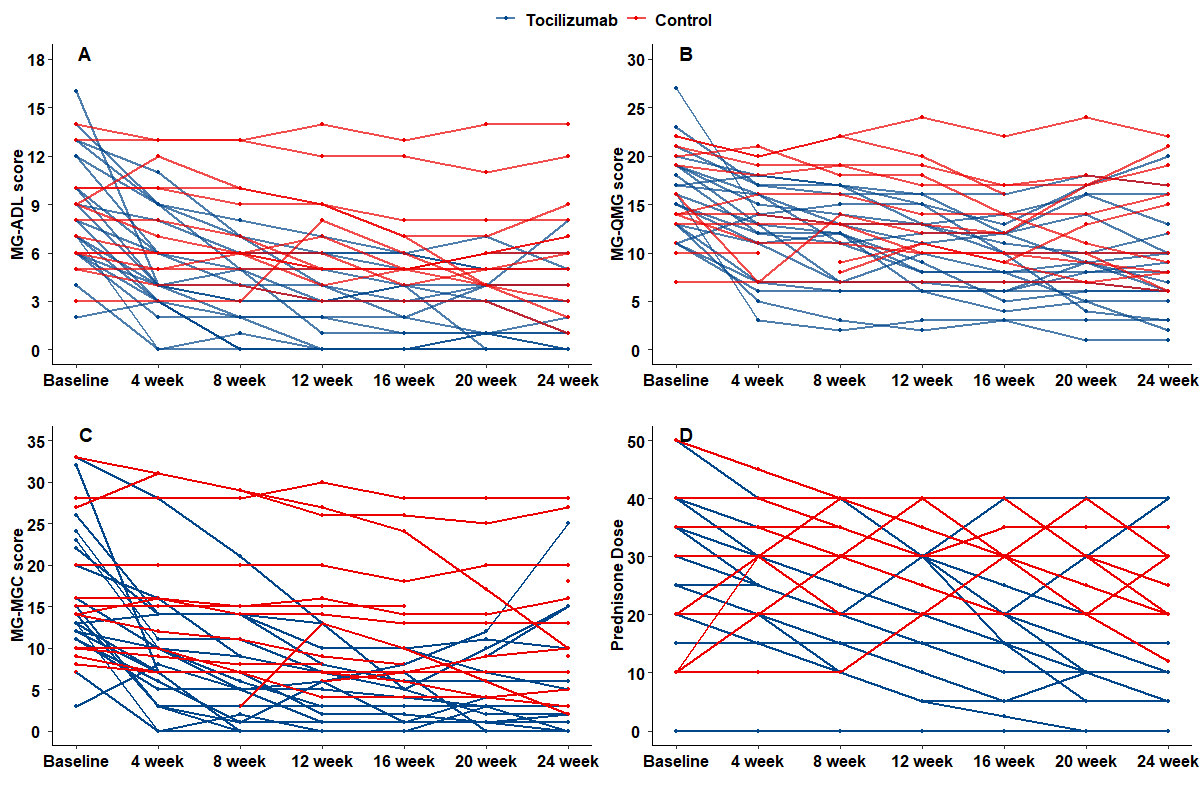
eFigure 1. Individual change of included 34 patients in the MG-ADL, QMG, and MGC scores and daily prednisone dose from baseline to 24 weeks**

The line charts show individual patient’s change in MG-ADL (A), QMG (B), MGC (C) scores and daily prednisone dose (D) form baseline to 24 weeks in the tocilizumab and control groups.

**
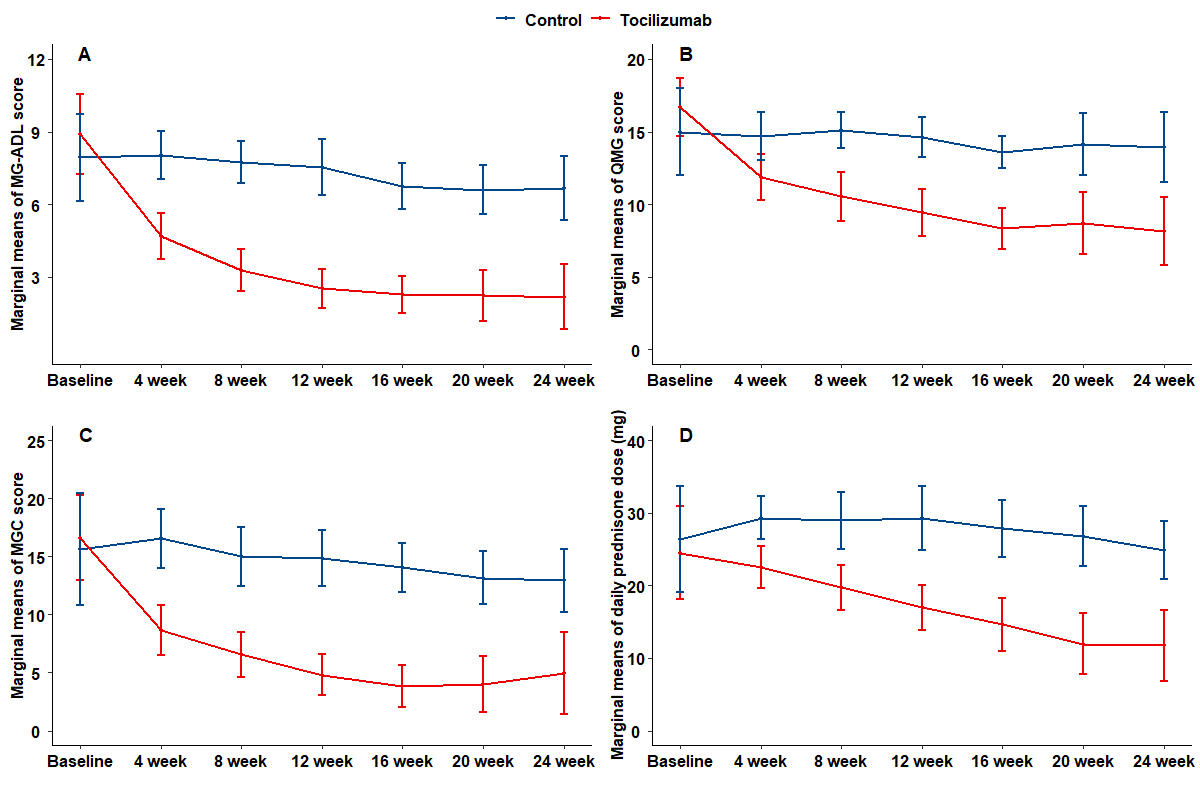
eFigure 2. Adjusted mean of MG-ADL (A), QMG (B), MGC (C) scores and daily prednisone dose changes from baseline to 24 weeks**

The line charts show adjusted mean change of all scores measure and the daily prednisone dose calculated by GEE model from baseline to 24 weeks in the tocilizumab and control groups.
